# Lipoma Arising in the Eustachian Tube

**DOI:** 10.7759/cureus.56597

**Published:** 2024-03-20

**Authors:** Takahiro Inoue, Takumi Kumai, Tomoki Yoshizaki, Miki Takahara

**Affiliations:** 1 Department of Otolaryngology-Head and Neck Surgery, Asahikawa Medical University, Asahikawa, JPN; 2 Department of Otolaryngology-Head and Neck Surgery, Asahikawa Kosei Hospital, Asahikawa, JPN

**Keywords:** eustachian tube, endoscopic sinus surgery, nasal congestion, nasopharynx, lipoma

## Abstract

Lipomas are among the most common soft tissue tumors. Surgical removal of lipoma is considered if the patient has symptoms or cosmetic challenges. Lipomas that develop from any fat tissue in the body and involve the eustachian tube are extremely rare.

Herein, we report the case of a patient with a lipoma arising in the eustachian tube. We also summarized the literature on tumors originating from the eustachian tubes.

A 62-year-old female presented to our department with a five-year history of left nasal congestion. Nasal endoscopy revealed a tumor in the left eustachian tube. The tumor was considered a lipoma on computed tomography (CT) and magnetic resonance imaging (MRI) and was removed using a transnasal endoscopic approach.

Nasal endoscopy and radiologic imaging can be used to detect tumors in the nasopharynx, including the eustachian tubes. Magnetic resonance imaging is particularly useful for the diagnosis of lipomas. A lipoma in the eustachian tube can cause nasal congestion and aural fullness, and the transnasal endoscopic approach is useful for tumor removal.

## Introduction

Lipoma is the most common benign tumor that develops from the mesenchymal connective tissue and is mostly asymptomatic [1]. Only 15% of lipomas are located in the head and neck region [2], mostly in the posterior neck [3]. Moreover, involvement of the eustachian tube in lipoma is extremely rare [4]. Herein, we report the case of a patient with a lipoma arising in the eustachian tube.

## Case presentation

**Figure 1:**
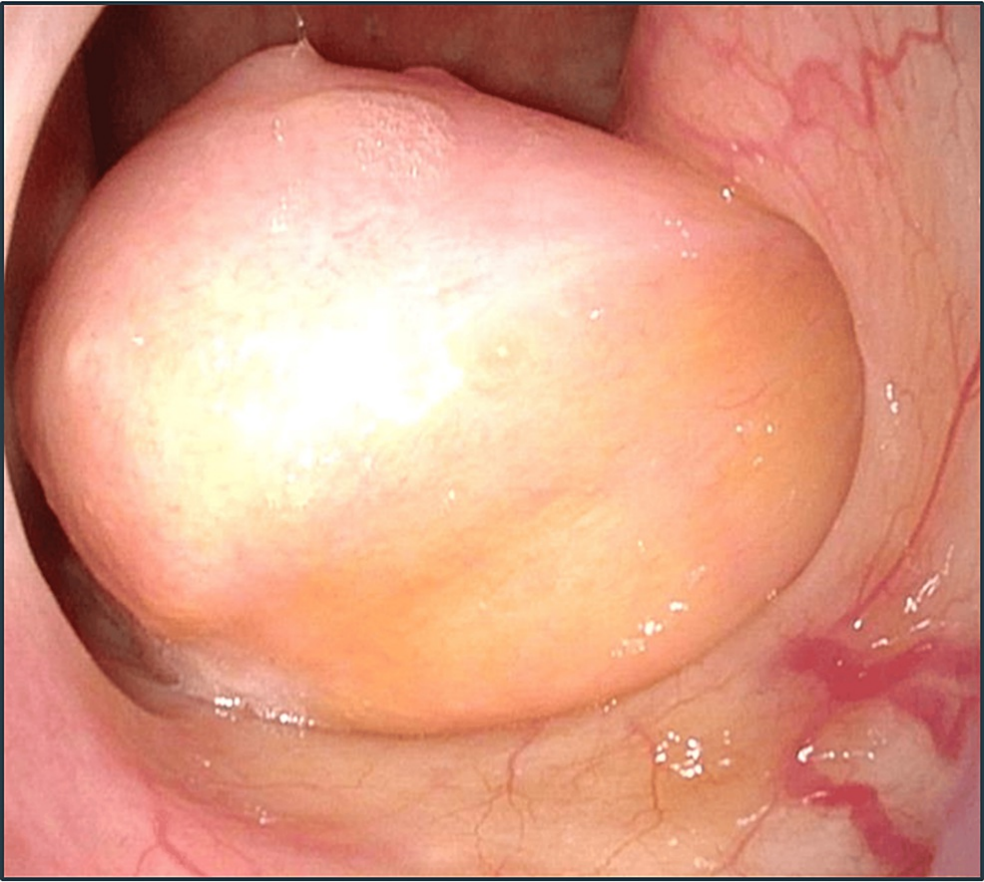
Finding of nasal endoscopy Nasal endoscopy revealed a smooth-surfaced tumor arising from the left eustachian tube to the nasopharynx (35 × 30 mm).

A 62-year-old female visited our department with a five-year history of left nasal congestion. Nasal endoscopy revealed a smooth-surfaced tumor that arose from the left eustachian tube to the nasopharynx (Figure 1).

**Figure 2:**
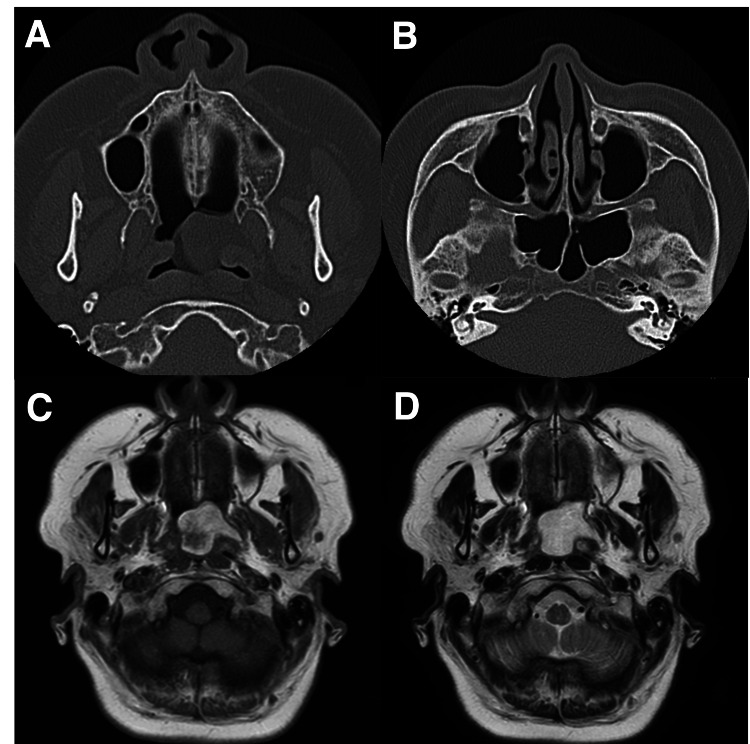
Findings of CT and MRI A: Axial plain CT scan revealed a tumor arising from the left eustachian tube with the same density as the surrounding fat. B: Axial plain CT scan revealed no evidence of OME. C: Axial plain T1-weighted MRI scan revealed a tumor with high signal intensity. D: Axial plain T2-weighted MRI scan revealed high signal intensity. CT: computed tomography, MRI: magnetic resonance imaging, OME: otitis media with effusion

The tympanic membrane showed partial myringosclerosis, but no clear middle ear effusion. No abnormal findings were observed in audiography and tympanometry. Computed tomography (CT) revealed a tumor protruding from the left eustachian tube into the nasopharynx, and no soft tissue shadow was observed in the middle ear, suggesting otitis media with effusion (OME). Magnetic resonance imaging (MRI) demonstrated that the tumor had high signal intensity in T1- and T2-weighted images (Figure 2).

**Figure 3:**
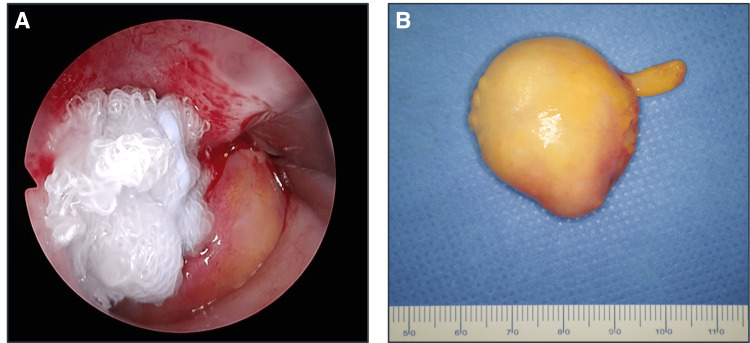
Intraoperative findings A: The tumor was pulled out into the nasopharynx, and the base of the tumor was excised. B: The tumor removed along with the base (35 × 30 mm).

Based on these findings, a lipoma was suspected, and surgical removal via a transnasal endoscopic approach under general anesthesia was performed. The tumor was completely embedded in the eustachian tube; however, it had good mobility. We pulled the tumor toward the nasopharynx and resected its base using scissors. The tumor was then removed from the oral cavity. No bleeding was observed after the resection. The patient was discharged without postoperative complications. The tumor was 35 × 30 mm (Figure 3).

**Figure 4:**
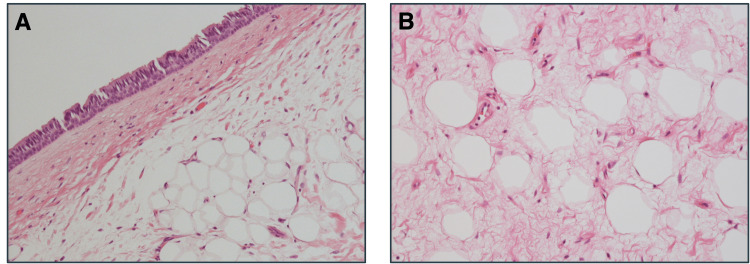
Histopathological findings A: Hematoxylin and eosin staining revealed that the epithelium consisted of a poorly atypical pseudostratified layer (original magnification, ×100). B: The tumor was composed of mature adipocytes with well-defined borders surrounded by a fibrous component (original magnification, ×200).

Histopathological examination revealed that the epithelium consisted of a poorly atypical pseudostratified epithelium composed of mature adipocytes with well-defined borders surrounded by fibrous components (Figure 4).

**Figure 5:**
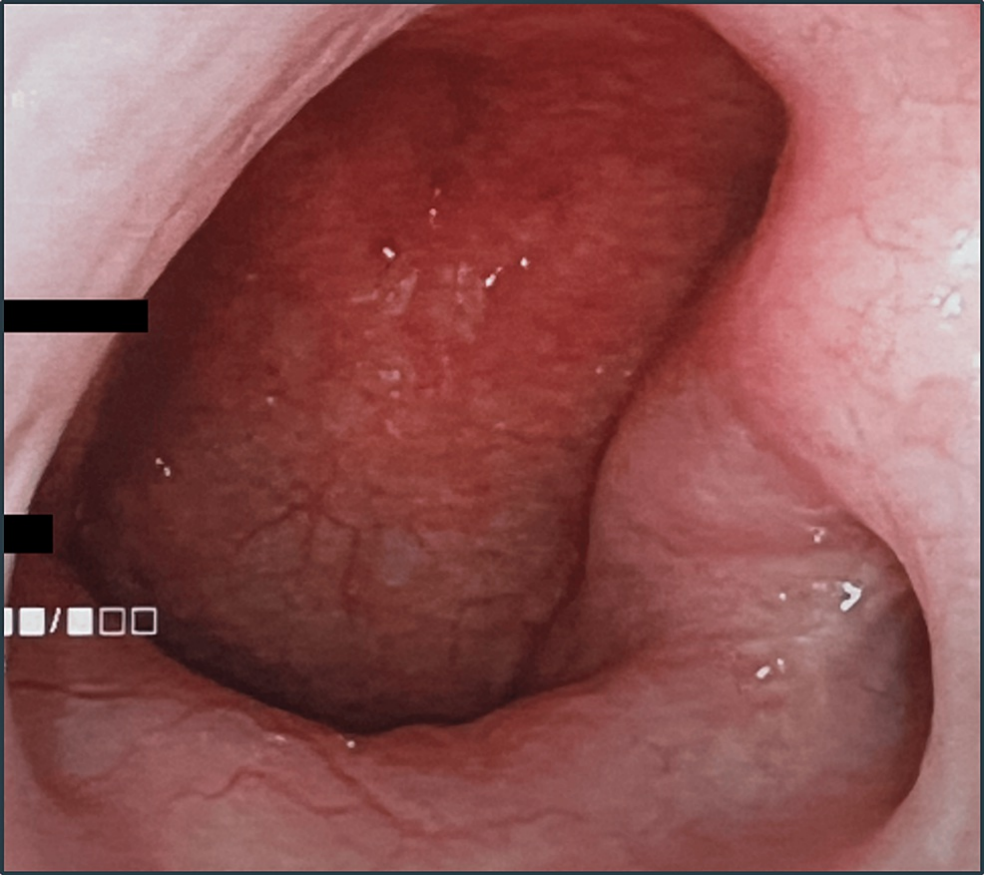
Findings of nasal endoscopy one year after the surgery Nasal endoscopy in the left nasal cavity revealed no recurrence.

There were no malignant findings, and the tumor was diagnosed as a lipoma. One year after surgery, no recurrence of the tumor or nasal obstruction symptoms were observed (Figure 5).

## Discussion

Tumors arising from the eustachian tube area are extremely rare. The symptoms induced by blocking the eustachian tube include aural fullness, tinnitus, otalgia, hearing loss, and nasal congestion. CT, MRI, nasal endoscopy, audiography, and tympanometry are required for the diagnosis. Although rare, various types of tumors in the eustachian tubes have been reported. We have summarized the reported cases of tumors arising from the eustachian tube (Table 1) [1,4-16].

**Table 1:** Summary of reported cases of tumor in the eustachian tube [1,4-16]

Tumor	Number
Carcinoma	21
Squamous cell carcinoma/transitional cell carcinoma	14
Mucoepidermoid carcinoma	6
Medullary carcinoma	1
Dermoid cyst	20
Melanin-pigmented oncocytic metaplasia	12
Lipoma	9
Teratoma	9
Melanoma	8
Chondroma	7
Sarcoma	3
Leiomyoma	1
Total	90

In a total of 90 cases, squamous cell carcinoma was the most common malignancy with 14 cases, followed by melanoma with eight cases. Among the benign tumors, dermoid cysts were the most common (20 cases), followed by melanin-pigmented oncocytic metaplasia (12 cases), and lipoma and teratoma (each with nine cases).

Lipomas are benign subcutaneous or submucosal tumors that occur between the ages of 50 and 60 years [17]. The origin of the lipoma in the eustachian tube could be the Ostmann fat pad, which is distributed along the eustachian tube. Lipomas are classified into various histological types, including fibro-, spindle cell, pleomorphic, myo-, angio-, chondroid, myxo-, and osteolipomas [8]. Liposarcoma must be considered if the tumor invades surrounding tissues [1]. Our case may be considered a fibrolipoma owing to a large number of fibrous components with mature adipocytes [18]. Imaging studies are useful for diagnosing lipomas. Because the densities of lipomas and fat are comparable on CT, MRI is more useful for diagnosing lipomas. MRI shows high signals in T1- and T2-weighted images, and the signal is suppressed on T1-weighted images with fat-suppressed contrast-enhanced MRI. Surgical removal involves the treatment of lipomas associated with cosmetic problems or subjective symptoms.

To date, nine cases of lipoma arising in the eustachian tube, including our case, have been reported (Table 2) [1,4-6,8,9,14,15].

**Table 2:** Summary of reported cases of lipoma in the eustachian tube OME: otitis media with effusion, +: with OME, -: without OME, N/A: not applicable [1,4-6,8,9,14,15]

Case	Year	Author	Age	Sex	Symptoms	OME	Tumor size (mm)	Follow-up (month)
1	2008	Park et al. [5]	42	Female	Aural fullness, hearing loss	+	10 × 10	3
2	2011	Liu et al. [4]	34	Female	Aural fullness, tinnitus, nasal congestion	+	16 × 24	5
3	2012	Fuji et al. [6]	29	Female	Nasal congestion	+	27 × 10	N/A
4	2013	Thakur et al. [8]	50	Female	Nasal congestion	-	40 × 40	6
5	2016	Aydin et al. [9]	43	Male	Otalgia, hearing loss	+	4 × 5	6
6	2016	Dabiri et al. [1]	47	Male	Aural fullness, otalgia	+	15 × 8	12
7	2022	Al Zaabi et al. [14]	27	Male	Aural fullness, tinnitus, hearing loss	+	10 × 8	36
8	2022	Ko et al. [15]	49	Female	Tinnitus, nasal congestion	+	7 × 7	12
9	2023	Present case	62	Female	Nasal congestion, aural fullness	-	35 × 30	12

Among these patients, 67% were female. The median patient age was 43 years (range: 27-62 years). The disease occurred on the right and left sides in six and three cases, respectively. Although not observed in the present case, OME was present in seven out of nine cases. The tumor in our case obstructed the eustachian tube; however, the patient did not develop OME, possibly because of the high mobility of the tumor. The median follow-up period after surgery was nine months (range: 3-36 years). Nasal congestion was the most common symptom, followed by aural fullness, hearing loss, tinnitus, and otalgia. Endoscopic sinus surgery (ESS) is a feasible minimally invasive approach to the eustachian tubes. All reported tumors, including our case, were removed using ESS, and none recurred.

## Conclusions

We report the case of a patient with a lipoma in the eustachian tube. Lipomas in the eustachian tube that cause symptoms such as nasal congestion and aural fullness may require surgical removal. The transnasal endoscopic approach is a feasible procedure to remove lipomas involving the eustachian tube.
